# RuvBL1 Maintains Resistance to TRAIL-Induced Apoptosis by Suppressing c-Jun/AP-1 Activity in Non-Small Cell Lung Cancer

**DOI:** 10.3389/fonc.2021.679243

**Published:** 2021-06-07

**Authors:** Hao Li, Taoran Zhou, Yue Zhang, Hengyi Jiang, Jing Zhang, Zichun Hua

**Affiliations:** ^1^ The State Key Laboratory of Pharmaceutical Biotechnology, School of Life Sciences, Nanjing University, Nanjing, China; ^2^ Changzhou High-Tech Research Institute of Nanjing University, Changzhou, China

**Keywords:** RuvBL1, TRAIL, c-Jun/AP-1, resistance, lung cancer, prognosis

## Abstract

Lung cancer is the common malignant tumor with the highest death rate in the world. Tumor necrosis factor-related apoptosis-inducing ligand (TRAIL) as a potential anticancer agent induces selective apoptotic death of human cancer cells. Unfortunately, approximately half of lung cancer cell lines are intrinsically resistant to TRAIL-induced cell death. In this study, we identified RuvBL1 as a repressor of c-Jun/AP-1 activity, contributing to TRAIL resistance in lung cancer cells. Knocking down RuvBL1 effectively sensitized resistant cells to TRAIL, and overexpression of RuvBL1 inhibited TRAIL-induced apoptosis. Moreover, there was a negative correlation expression between RuvBL1 and c-Jun in lung adenocarcinoma by Oncomine analyses. High expression of RuvBL1 inversely with low c-Jun in lung cancer was associated with a poor overall prognosis. Taken together, our studies broaden the molecular mechanisms of TRAIL resistance and suggest the application of silencing RuvBL1 synergized with TRAIL to be a novel therapeutic strategy in lung cancer treatment.

## Introduction

Lung cancer is the most common malignant tumor with high death rate. Up to 85% of lung cancer cases are non-small cell lung cancer (NSCLC). For patients with NSCLC, the 5−year survival rate is 55% at an early stage, but reduces to <5% with distant metastasis (1). Many lung cancer cells can escape apoptosis and develop the resistance to radiation- or chemo-therapy (2, 3). Thus, deep-understanding on the molecular mechanism of developing the resistance to apoptotic cell death is urgently needed in lung cancer therapy.

Tumor necrosis factor–related apoptosis-inducing ligand (TRAIL), is a potential cancer therapeutic agent, which selectively triggers apoptosis in tumor cells not in normal cells (4). TRAIL-related drugs were clinically carried out in lung cancer, colorectal cancer, breast cancer, and lymphoma (5, 6). However during the process of treatment, tumor cells can acquire TRAIL resistance to escape apoptotic cell death induced by TRAIL (7, 8). Preclinical studies indicate that most of NSCLC cells are intrinsically resistant to TRAIL-induced cell death (9). Abnormal expressions of anti-apoptotic proteins such as BCL-2, BCL-xL and c-FLIP, resulted in resistance to TRAIL in NSCLC (10, 11). Also, activations of PI3K/Akt and NF-κB pathway were also associated with TRAIL-resistance in NSCLC (12, 13). Nonetheless, the mechanism of developing TRAIL resistance is not fully clarified. Further investigation on the enhancement of TRAIL-sensitivity is required for improving the therapeutic potential in lung cancer.

RuvBL1 (synonyms: Pontin52, Rvb1, TAP54α, and TIP49) is a highly conserved ATPase of the AAA+ (ATPases Associated with various cellular Activities) superfamily, and possesses intrinsic ATPase activities and DNA helicase activities (14–16). It is involved in various cellular processes, such as transcription, DNA damage, cellular transformation and cancer metastasis (17–19). Increased expression of RuvBL1 was found in lung, gastric, breast, kidney and leukemic cancers, and correlated with a poor prognosis (20). In particular, several studies suggested a potential use of RuvBL1 as a biomarker for diagnosis and prognosis of lung cancer (21). High expression of RUVBL1/2 in NSCLC patient tumors are associated with poor survival. Pharmacological inhibition of RUVBL1/2 resulted in modest antitumor activity and synergized with radiation in NSCLC (22). Consistent with poor prognosis, RuvBL1 was previously reported in the regulation of apoptosis. Knockdown of RuvBL1 in HCC cells led to spontaneous apoptosis (23). RuvBL1 with its ATPase activity modulated the apoptotic activity of c-Myc and of E2F1 (24). In addition, RuvBL1 negatively regulates JNK-mediated cell death in Drosophila (25). Therefore, we speculate that RuvBL1 overexpression might be associated with TRAIL-resistance in NSCLC, and downregulation of RuvBL1 probably sensitizes lung cancer cells to TRAIL-induced apoptosis.

In this study, we reported that RuvBL1 expression was positively related to TRAIL resistance in NSCLC. Overexpression of RuvBL1 significantly inhibited TRAIL-induced apoptosis by downregulating c-Jun/AP-1 activity. These observations suggested that RuvBL1 might be a promising target for TRAIL-based therapy against lung cancers.

## Materials and Methods

### Reagents

Recombinant human TRAIL was obtained from Peprotech (Rocky Hill, NJ). Specific antibodies against RuvBL1, caspase-3, cleaved caspase-3, c-Jun, c-Fos, Flag-tag, phosho-JNK, JNK, phospho-ERK1/2 and ERK were obtained from Cell Signaling Technology (Danvers, MA). RuvBL1 polyclonal antibody (PA5-29278, Thermo Scientific™) and CHIP kit (#26156, Pierce) were purchased from Invitrogen.

### Cell Culture and Transfection

NSCLC cells (A549, H1299, H446, SPC, and SPCA1) were obtained from the Type Culture Collection of the Chinese Academy of Sciences (Shanghai, China) and maintained in RPMI−1640 medium supplemented with 10% fetal bovine serum (Hyclone, Ltd., USA), 100 units/ml penicillin, and 100 μg/ml streptomycin at 37°C in 5% CO2. Cell transfection was performed by Lipofectamine 2000 (Invitrogen) according to the instructions of the manufacturer.

### Cell Viability and Cell Death Assays

Cell viability was detected by Cell Counting Kit (CCK-8, Dojindo Ltd, Japan). In briefly, cells were seeded in a 96-well plate and treated with TRAIL (0-100ng/ml) for 6h, then incubated with CCK-8 solution reagent for 2h. The optical density (OD450 nm) was measured by a microplate reader (Rayto Ltd., China).

For apoptosis detection, cells were digested with EDTA-free trypsin and harvested the single cell suspension. After washing with binding buffer, cells were stained with Annexin V−FITC and propidium iodide (PI) using Annexin V-FITC/PI Apoptosis Detection Kit (Beyotime, Shanghai) according to the manufacturer’s protocol. Apoptotic analysis was performed by Accuri C6 FACScan flow cytometer using CellQuest software (Becton Dickinson, Franklin Lakes, NJ, USA).

### Western Blotting

The cells were washed with PBS and lysed in RIPA buffer on ice. The supernatant was collected by centrifuge at 10,000 rpm for 10 min at 4°C. Equal quantity of protein samples was loaded and separated on 12% SDS-PAGE gels before transferring to PVDF membranes (Millipore, Danvers, MA). After blocking in 5% skim milk in TBST, the membranes were incubated with primary antibodies at 4°C overnight. Then the membranes were washed three times in TBST for 5 min each, and incubated with HRP-conjugated secondary antibodies. The protein bands were visualized using an enhanced chemiluminescence (ECL) kit (Thermo Scientific, Inc) and were quantified using ImageJ software.

### AP-1 Luciferase Assay

AP-1 luciferase reporter was provided by Dr. Nancy H Colburn (National Cancer Institute, Frederick, MD) and reporter gene assays were carried out as described previously. In brief, cells seeded in 24-well plates were cotransfected with AP-1 luciferase reporter plasmids (0.2μg) together with Renilla reporter pRL-null as an internal control (0.1μg), and expressing vector as indicated (0.1–0.5μg). The total amount of DNA for each transfection kept constant. After 24h transfection, cells were harvested for luciferase assays using Dual-Luciferase reporter assay system (E1910, Promega) following the manufacturer’s instructions. The measured luciferase activities were normalized to Renilla reporter expression.

### ChIP Assay

Chromatin immunoprecipitation (ChIP) assay is a powerful tool to study protein-DNA interaction. Pierce™ agarose ChIP kit was used in the study according to the manufacturer’s protocol. To evaluate the binding of c-Jun to AP-1 sites in the promoter, cell lysate was immunoprecipitated with anti-c-Jun antibody, using anti-RNA polymerase II antibody as positive control and normal rabbit IgG as negative control. After IP elution and DNA recovery, 20μl of each resulting solution of purified DNA was analyzed by qPCR detection using a pair of primers that flank the AP-1 binding site in the *c-jun* promoter. The sequences were as follows: the AP-1 site in *c-jun* promoter, 5’-GAACTGCCTTCAGAGCCA and 5’- CACTTATCCTCACCTCCCTC. A negative control primer of *c-jun* coding region: 5’- CCTTGAAAGCTCAGAACTCGGAG and 5’- TGCTGCGTTAGCATGAGTTGGC.

### Oncomine Analyses

Oncomine is a cancer microarray database and web-based data-mining platform (http://www.oncomine.org). Expression analyses for RuvBL1 and c-Jun were performed in Oncomine by primary filters with “cancer *vs* normal analysis” and “lung cancer”. Datasets are threshold by P-value of 1E-4, Fold-change of 2, and Gene rank percentile of top 10%. Hou lung cancer dataset is ranked on RuvBL1 overexpression with the value of p=3.39E-11 and fold change=2.121. There are 65 adjacent normal lung tissue samples and 91 tumor samples (19 large cell lung carcinomas, 27 squamous cell lung carcinomas and 45 lung adenocarcinomas) in Hou lung cancer dataset (26). Raw data of lung adenocarcinomas (n=45) was downloaded for the correlation expression analysis of RuvBL1 and c-Jun by linear fitting.

### Statistical Analysis

Statistical analyses were conducted with Student’s t test (unpaired two-tailed, unequal variance). All experiments were performed at least 3 times. The experimental data were processed by Graphpad Prism 6.0 and presented as mean ± SD. Significance level was set at *, P < 0.05; **, p < 0.01.

## Results

### RuvBL1 Expression Contributes to TRAIL Resistance in Lung Cancer Cells

**Figure 1:**
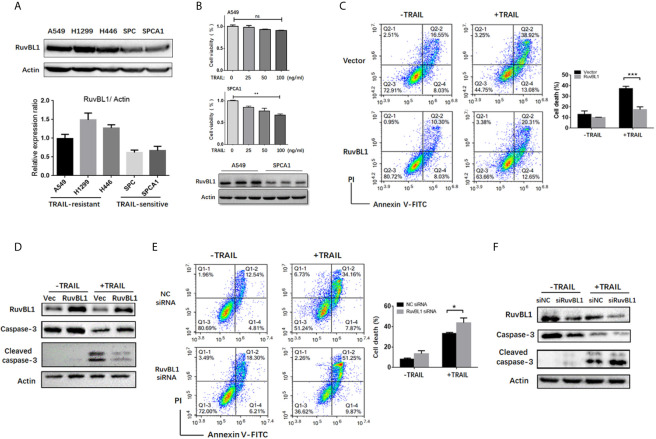
RuvBL1 expression correlates with TRAIL resistance in lung cancer cells. **(A)** Western blots for RuvBL1 expression in lung cancer cell lines, which were divided into two groups: TRAIL -resistance and TRAIL-sensitive. By gray scale values, relative levels of RuvBL1 normalized to Actin were shown as mean ± SD representative of three independent experiments. **(B)** Cell viability for A549 and SPCA1 in the treatment with TRAIL (0-100ng/ml) for 8h was measured by CCK8 assay. Data shown as mean ± SD. **P < 0.01; ns, non-significance. Western blot analysis of RuvBL1 expression in the bottom. **(C)** A549 cells transfected with empty or RuvBL1 vector were treated with TRAIL (50 ng/ml) for 12 h. Cell death was detected by Annexin V/PI staining. Quantitative analysis of double positive cells was shown as mean ± SD (n=3). ***P < 0.001. Western blots for procaspase-3, cleaved capase-3 and RuvBL1 were shown in **(D)**. **(E)** A549 cells transfected with negative control siRNA (siNC) or RuvBL1 siRNAs (siRuvBL1) were treated with TRAIL (50 ng/ml) for 12 h. Cell death was detected by Annexin V/PI staining. Quantitative analysis of double positive cells was shown as mean ± SD (n=3). *P < 0.05. Western blots for procaspase-3, cleaved capase-3 and RuvBL1 were shown in **(F)** representative of three independent experiments.

To examine the possibility of RuvBL1 implicated in TRAIL resistance, differential resistance of lung cancer cell lines to TRAIL were used for the study. A549, H1299 and H446 cells were resistant to TRAIL (9), and conversely, SPC and SPCA1 as TRAIL-sensitive cells. Western blot analysis showed that RuvBL1 expression was higher in TRAIL-resistant cells and lower in TRAIL-sensitive cells (**Figure 1A**). Consistent with the resistant to TRAIL, CCK8 assays showed that SPCA1 cells were sensitive to TRAIL-induced cell death in a dose-dependent manner, while A549 cells were maintained in approximately 90% cell viability (**Figure 1B**), suggesting a positive correlation of RuvBL1 and TRAIL resistance.

To validate the effect of RuvBL1 against TRAIL-induced apoptosis, A549 cells were overexpressed with RuvBL1 and exposed to TRAIL. RuvBL1 overexpression significantly inhibited TRAIL-induced apoptosis by FACS analysis (**Figure 1C**). Consistently, activation of caspase-3 was also reduced in TRAIL-treated cells when RuvBL1 overexpression (**Figure 1D**). Similar results were observed in SPCA1 cells (**Supplementary Figure S1**). Furthermore, knockdown of RuvBL1 promoted TRAIL-induced cell death and enhanced caspase-3 activation in A549 cells (**Figures 1E, F**). These results indicate that RuvBL1 protects lung cancer cells against TRAIL-induced cell death.

### RuvBL1 Inhibits TRAIL-Induced Apoptosis by Downregulation of c-Jun

**Figure 2:**
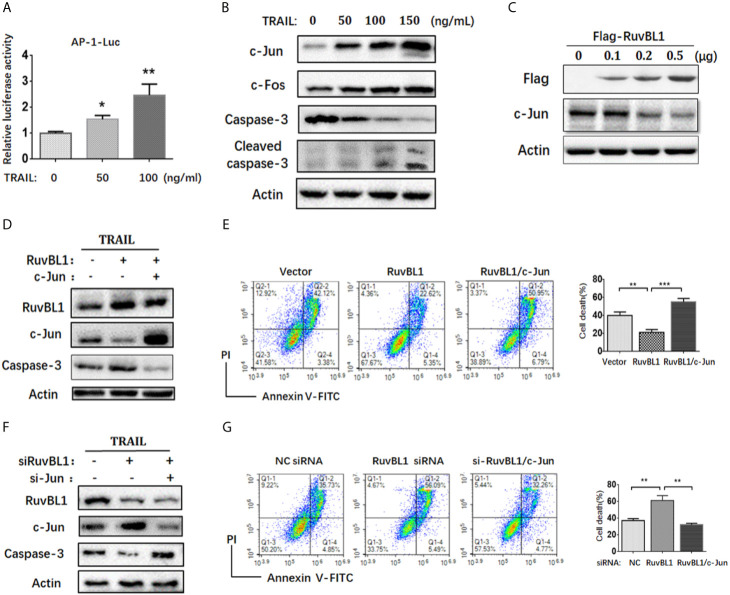
RuvBL1 inhibits TRAIL-induced apoptosis *via* downregulation of c-Jun. **(A)** Reporter gene assays for AP-1 activity in A549 cells co-transfected with AP-1-luc reporter and Renilla reporter in the treatment with TRAIL (0-100ng/ml) for 12h. The fold-change of AP-1 luciferase activity *versus* Renilla shown as mean ± SD (n=3). *P < 0.05; **P < 0.01 **(B)** Western blots for c-Jun, c-Fos, caspase-3 and cleaved caspase-3 in A549 cells treated with TRAIL (0-150ng/ml) for 12h. **(C)** A549 cells were transfected with an increasing amount of Flag-RuvBL1 expression vector (0-0.5μg) for 36h. The expression of Flag-RuvBL1 and c-Jun were examined by western blotting. **(D)** Western blots for caspase-3, RuvBL1 and c-Jun expression in A549 cells transfected with empty vector, or Flag-RuvBL1, or Flag-RuvBL1 plus Flag-c-Jun respectively followed by treatment with TRAIL (50 ng/ml) for 12 h. Annexin V/PI staining by FACS analysis shown in **(E).** Cell death shown as mean ± SD (n=3). **P < 0.01; ***P < 0.001. **(F)** Western blots for caspase-3, RuvBL1 and c-Jun expression in A549 cells transfected with siRNAs followed by the treatment with TRAIL (50 ng/ml) for 12 h. Cell death by Annexin V/PI staining in **(G)** was shown as mean ± SD (n=3). **P < 0.01, representative of three independent experiments.

Activator protein 1 (AP-1) is a key transcription factor responsible for cell survival proliferation and cell death. Previous reports pointed out an essential role of AP-1 activity in TRAIL-sensitive cancer cells (27–29). Consistent with these reports, the increased AP-1 activities were also observed in A549 cells treated with TRAIL (**Figure 2A**). Upon TRAIL treatment, c-Jun and c-Fos, as main AP-1 family members, were both up-regulated, especially the increased c-Jun protein closely related to increased apoptosis (**Figure 2B**). RuvBL1 has been reported to regulate transcription through interaction with MYC, β-catenin-LEF/TCF, and E2F (24, 30). To test the possible regulation of RuvBL1 on c-Jun expression, we examined the levels of c-Jun with an increasing expression of RuvBL1. Excitingly, RuvBL1 overexpression actually reduced the c-Jun level in a dose-dependent manner (**Figure 2C**). We further assessed the effect of c-Jun on the inhibition of TRAIL-induced apoptosis by RuvBL1 overexpression. As expected, RuvBL1 overexpression reduced the level of c-Jun and caspase-3 activation, and the recovery expression of c-Jun restored the cell death induced by TRAIL (**Figures 2D, E**). Similar results were also obtained in SPCA1 cells (**Supplementary Figure S2**). Further confirmation was performed by knockdown of RuvBL1 and c-Jun. Consistently, RuvBL1 knockdown led to the upregulation of c-Jun and caspase-3 activation, and c-Jun siRNAs inhibited the increased apoptosis by FACS analysis (**Figures 2F, G**). These data suggest that RuvBL1 inhibits TRAIL-induced apoptosis through downregulation of c-Jun.

### RuvBL1 Inhibits AP-1 Transcriptional Activity

**Figure 3:**
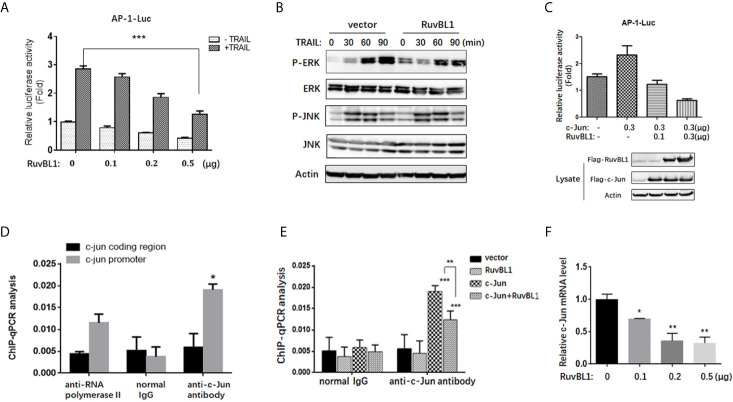
RuvBL1 inhibits AP-1 transcriptional activity. **(A)** Reporter gene assays for AP-1 activity in A549 cells with an increasing expression of Flag-RuvBL1 in the treatment with TRAIL (100 ng/ml) for 12h. The fold-change of AP-1 luciferase activity shown as mean ± SD (n=3). ***P < 0.001. **(B)** Western blots for p-ERK/total ERK, p-JNK/total JNK in A549 cells treated with TRAIL (100 ng/ml) for different times. **(C)** Reporter gene assays for AP-1 activity in A549 cells with co-transfection of Flag-c-Jun and RuvBL1 as indicated. Western blots for c-Jun and RuvBL1 expression shown in the below. **(D)** A549 cells with c-Jun overexpression were immunoprecipitated with antibodies as indicated for ChIP assay. qPCR for ChIP DNA with two pairs of primers, one for the AP-1 site in *c-jun* promoter, another for the coding region of *c-jun* as negative control. **(E)** A549 cells were transfected with c-Jun, or RuvBL1, or c-Jun plus RuvBL1 respectively. ChIP assay using antibodies as indicated. ChIP DNA was analyzed by qPCR with a pair of primer of the AP-1 site in *c-jun* promoter. Data shown as mean ± SD (n=3). **P < 0.01. **(F)** QPCR for *c-jun* mRNA in A549 cells with RuvBL1 overexpression. Data was shown as mean ± SD of three independent experiments. *P < 0.05; **P < 0.01 *versus* control group.

To address the molecular mechanism of RuvBL1 on regulating c-Jun expression, we firstly examined the effect of RuvBL1 on AP-1 activity by AP-1 luciferase reporter in A549 cells. Reporter gene assays showed that RuvBL1 overexpression significantly decreased AP-1 luciferase activity in TRAIL-treated cells (**Figure 3A**). AP-1 activity responds to extracellular stimulation mainly *via* JNK and ERK pathways (31). However, RuvBL1 overexpression did not affect JNK and ERK activation induced by TRAIL (**Figure 3B**). For no impact on JNK and ERK signaling in the cytoplasm, we speculate that AP-1 activity might be regulated by RuvBL1 in the nucleus. C-Jun is a predominant protein of AP-1 family and activates AP-1 transcriptional activity in the form of Jun/Jun homodimer or Jun/Fos heterodimer (32). With a high affinity for AP-1 site, c-Jun overexpression induced the AP-1 activation, but the increase on AP-1 activity was inhibited by RuvBL1 overexpression (**Figure 3C**), suggesting the involvement of RuvBL1 on AP-1 transcription regulation. Further validation by ChIP assay was performed on *c-jun* promoter, because *c-jun* gene contains two AP-1 sites in its own promoter (33). DNA binding of c-Jun protein was observed on *c-jun* promoter not on *c-jun* coding region. The specificity on Ch-IP assay with anti-Jun antibody was verified by comparison of normal IgG as negative control and anti-Polymerase II antibody as positive control (**Figure 3D**). As expected, RuvBL1 overexpression suppressed the DNA binding activity of c-Jun/AP-1 proteins (**Figure 3E**), and reduced the level of c-Jun mRNA in A549 cells (**Figure 3F**). These data suggested that high expression of RuvBL1 resulted in the decreased c-Jun level by inhibiting c-Jun/AP-1 transcriptional activity, contributing to TRAIL resistance.

### The Negative Correlation Expression of RuvBL1 and c-Jun in Lung Cancer

**Figure 4:**
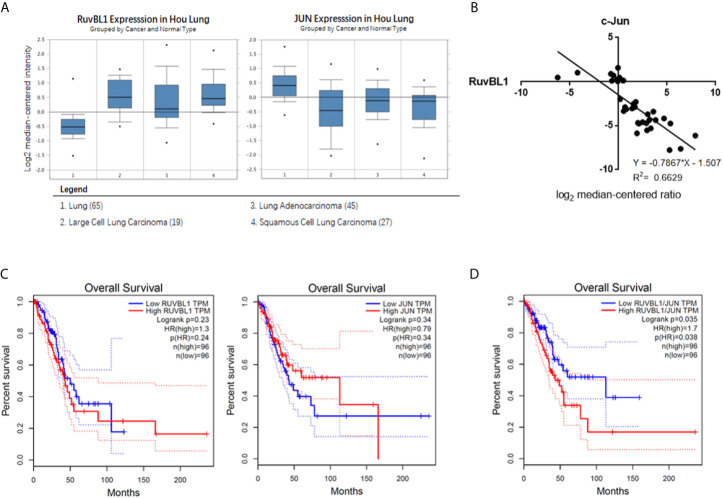
The negative correlation expression of RuvBL1 and c-Jun in lung adenocarcinoma **(A)** The meta-analysis of RuvBL1 and c-Jun expression in Oncomine. Hou Lung dataset contains 65 adjacent normal lung tissue samples and 91 lung tumor samples. **(B)** Regression analysis of the correlation between RuvBL1 and c-Jun expression in lung adenocarcinoma from Hou Lung dataset. **(C)** Kaplan Meier plotter for RuvBL1 or c-Jun expression on the survival rate of patients with lung adenocarcinoma. **(D)** Kaplan Meier plotter for the expression pattern of RuvBL1 *versus* c-Jun. The expression pattern of RuvBL1/c-Jun at high ratio is significantly associated with a worse prognosis. P < 0.05.

To investigate the clinical relevance of RuvBL1 and c-Jun in lung cancer, we analyzed their expression correlation by Oncomine analysis. Through threshold by P-value of 1E-4, Fold-change of 2, and Gene rank percentile of top 10%, Hou Lung dataset is selected in preference (26). In Hou lung dataset, higher level of RuvBL1 and lower level of c-Jun are present in lung tumor samples (n=91) compared with normal lung tissue samples (n=65) (**Figure 4A**). Through the regression analysis, the increased RuvBL1 was linearly related to the decreased c-Jun in lung adenocarcinoma, indicating a negative correlation expression of RuvBL1 and c-Jun (**Figure 4B**). Using GEPIA (http://gepia.cancer-pku.cn/index.html), a web server based on TCGA for cancer gene interactive analyses, Kaplan plots were drawn for RuvBL1 and c-Jun in the overall survival of patients with lung adenocarcinoma (LUAD dataset). Analysis of either RuvBL1 or c-Jun alone was not significantly correlated with clinical outcome (**Figure 4C**). But considering the expression patterns of RuvBL1 and c-Jun together, the relative expression of RuvBL1/Jun at high ratio was significantly associated with a worse survival. The expression pattern of low RuvBL1 and high c-Jun at low ratio of RuvBL1/c-Jun led to a survival benefit (**Figure 4D**). Therefore, the clinical relevance of RuvBL1 and c-Jun in lung cancer supports our results that RuvBL1 overexpression maintains TRAIL resistance by downregulating c-Jun.

## Discussion

TRAIL is a potential antitumor agent because it selectively induces apoptosis in tumor cells, not in normal cells. However, preclinical studies indicated that a considerable number of NSCLC develop resistance to TRAIL (9). The resistance to TRAIL is due to abnormal expression of anti-apoptotic proteins and dysfunction of TRAIL receptors (10–13). Here, we report that RuvBL1 is highly expressed in lung cancer and promotes the resistance to TRAIL.

RuvBL1 is implicated in various cellular processes related to oncogenesis. RuvBL1 associates with proteins involved in cell proliferation, cell cycle and DNA damage repair (20). But less investigation is focused on the role of RuvBL1 in apoptosis. In our study, RuvBL1 was highly expressed in TRAIL-resistance lung cancer cells compared to TRAIL-sensitive cells. Downregulation of RuvBL1 sensitized cells to TRAIL-induced apoptosis, and improved the efficacy of TRAIL in TRAIL-resistant lung cancer cells (**Figure 1**). Mechanical investigation indicated RuvBL1 as an anti-apoptotic molecule inhibited c-Jun/AP-1 transcriptional activity. RuvBL1 is known as a transcription cofactor to modulate the transcriptional activities of MYC, β-catenin and p53 (17). This is the first time to reveal the negative regulation of RuvBL1 on AP-1 transcriptional activity. RuvBL1 did not affect TRAIL-induced JNK and ERK activation in the cytoplasm, but RuvBL1 regulated c-Jun/AP-1 transcriptional activity by suppressing its DNA binding ability in the nucleus.

AP-1 activity is closely related to tumor development and targeting AP-1 is an attractive therapeutic strategy for cancer therapy. Previous studies reported that AP-1 activity was increased in TRAIL-sensitive cells and upregulation of c-Jun augmented TRAIL-mediated apoptosis. For examples, MG-132 increased the level of c-Jun/AP-1 and combination of MG-132 with TRAIL promoted cell death in TRAIL-resistant prostate cancer cells (28). Inhibition of AP-1 by knockdown of c-Jun blocked DR4 expression and attenuated TRAIL-induced apoptosis (29). Consistently, we found that TRAIL induced the increased AP-1 activity together with the upregulations of c-Jun and c-Fos in A549 cells. Downregulation of c-Jun increased the resistance of cells to TRAIL, suggesting that c-Jun is key regulator to increase the sensitivity to TRAIL (**Figure 2**). The inhibition of RuvBL1 on TRAIL-induced apoptosis was due to the downregulation of c-Jun (**Figure 2**). Furthermore, we demonstrated that RuvBL1 overexpression reduced the level of c-Jun by inhibiting AP-1 activity (**Figure 3**). Most importantly, there is a negative correlation expression between RuvBL1 and c-Jun in lung cancer tissues, supporting our conclusion that RuvBL1 is a negative regulator of c-Jun (**Figure 4**). In addition, the expression pattern of high RuvBL1 and low c-Jun was associated with a worse survival in lung adenocarcinoma, as reported that RuvBL1 is a potential prognosis biomarker in lung cancer. Further light into the role of RuvBL1 in a poor prognosis helps to develop novel therapeutics for NSCLC.

In conclusion, we demonstrate, for the first time, that RuvBL1 plays an anti-apoptotic role in resistance to TRAIL. Our results describe RuvBL1 as a negative regulator of c-Jun, and provide a possible mechanism of TRAIL resistance *via* RuvBL1 and c-Jun/AP-1. Although the molecular mechanism of RuvBL1 on regulating c-Jun/AP-1 activation is not fully understood, our studies suggest RuvBL1 to be a potential therapeutic target for TRAIL resistance in NSCLC, which helps to broaden the application of TRAIL.

## Data Availability Statement

The original contributions presented in the study are included in the article/**Supplementary Material**. Further inquiries can be directed to the corresponding authors.

## Author Contributions

HL and TZ: Investigation and data curation. YZ: Formal analysis and validation. JZ: Investigation and original draft preparation. ZH: Funding acquisition and resources. All authors contributed to the article and approved the submitted version.

## Funding

This study was supported in part by grants from the National Nature Science Foundation of China (81630092), National Key R&D program (2017YFA0506002), Jiangsu Provincial Department of Science and Technology (BK20192005, BK20171202).

## Conflict of Interest

The authors declare that the research was conducted in the absence of any commercial or financial relationships that could be construed as a potential conflict of interest.
