# Supplementary material for: RuvBL1 Maintains Resistance to TRAIL-Induced Apoptosis by Suppressing c-Jun/AP-1 Activity in Non-Small Cell Lung Cancer
Source: Front Oncol. 2021 Jun 7;11:679243. doi: 10.3389/fonc.2021.679243 (PMC8215499; doi:10.3389/fonc.2021.679243)
Supplement: Supplementary file 1 [file DataSheet_1.pdf]

Supplementary data:

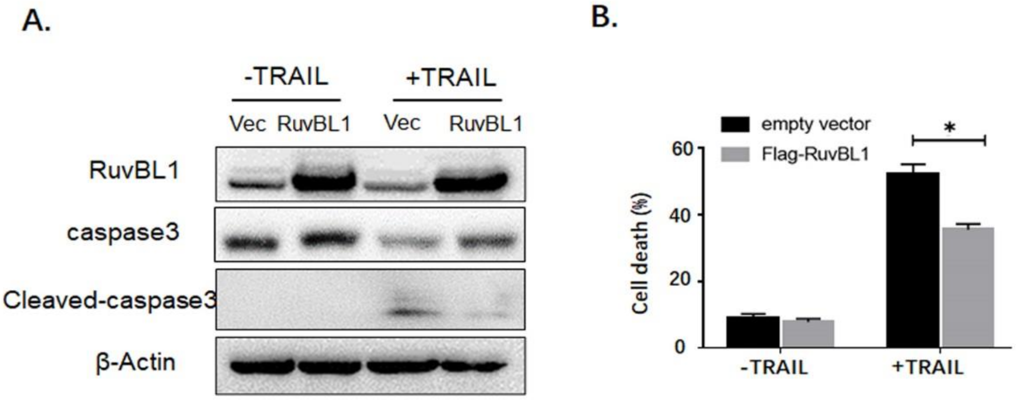

Figure S1. RuvBL1 overexpression inhibits TRAIL-induced apoptosis in SPCA1 cells. SPCA1 cells transfected with empty or RuvBL1 vector were treated with TRAIL (100 ng/ml) for 6 h. **(A)** Western blots for procaspase-3, cleaved capase-3 and RuvBL1 expression. **(B)** Cell death was detected by Annexin V/PI staining. Quantitative analysis of double positive cells was shown as mean ± SD. * $P < 0.05$.

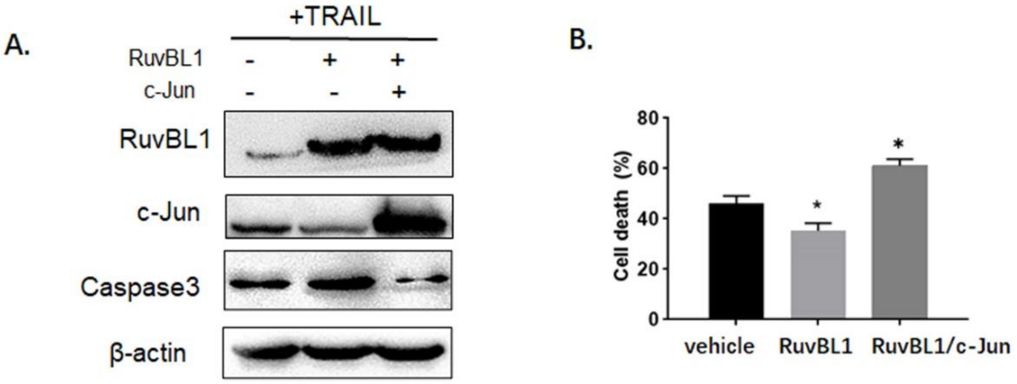

Figure S2. C-Jun overexpression increased TRAIL-induced apoptosis in SPCA1 cells. SPCA1 cells were transfected with empty vector, or Flag-RuvBL1, or Flag-RuvBL1 plus Flag-c-Jun respectively. After 24h transfection, cells were treated with TRAIL (100 ng/ml) for 6 h. **(A)** Western blots for procaspase-3, c-Jun and RuvBL1 expression. **(B)** Cell death was detected by Annexin V/PI staining. Quantitative analysis of double positive cells was shown as mean ± SD. * $P < 0.05$.
